# The promise of mitochondria in the treatment of glioblastoma: a brief review

**DOI:** 10.1007/s12672-025-01891-y

**Published:** 2025-02-09

**Authors:** Zhuo Liang, Songyun Zhao, Yuankun Liu, Chao Cheng

**Affiliations:** Department of Neurosurgery, The Affiliated Wuxi People’s Hospital of Nanjing Medical University, Wuxi, China

**Keywords:** Glioblastoma, Mitochondria, Oxidative phosphorylation, ROS, Mitochondrial metastasis, Mitochondrial autophagy

## Abstract

Glioblastoma (GBM) is a prevalent and refractory type of brain tumor. Over the past two decades, there have been minimal advancements in GBM therapy. The current standard treatment involves surgical excision followed by radiation and chemotherapy. Compared to other tumors, GBM is more challenging to treat due to the presence of glioma stem-like cells (GSCs) and the blood–brain barrier, resulting in an extremely low survival rate. Mitochondria play a critical role in tumor respiration, metabolism, and multiple signaling pathways involved in tumor formation, progression, and cell apoptosis. Consequently, mitochondria represent promising targets for developing novel anticancer agents, including those targeting oxidative phosphorylation, reactive oxygen species (ROS), mitochondrial transfer, and mitophagy. This review outlines the mitochondrial-related therapeutic targets in GBM, highlighting the potential of mitochondria as a target for GBM treatment.

## Introduction

Gliomas constitute nearly 30% of primary brain tumors and 80% of all malignant brain tumors [1]. In 2021, the WHO updated its classification of central nervous system tumors, incorporating genetic features and molecular patterns alongside histopathological analyses, including multi-omics approaches, to classify different types of gliomas. Isocitrate dehydrogenase (IDH) has long been known to catalyze the oxidative decarboxylation of isocitric acid to produce α-ketoglutarate (αKG) and carbon dioxide (CO2), as well as the reduction of the cofactor NAD(P)^+^ to NAD(P)H. The role of IDH includes the regulation of mitochondrial oxidative phosphorylation, glutamine metabolism, adipogenesis, and cellular redox state [2, 3]. IDH-wildtype diffuse gliomas are now recognized as GBMs [4, 5]. GBM is the most prevalent and aggressive form of primary brain tumor, accounting for up to 50% of all gliomas [6]. Despite first-line treatment for GBM, which includes maximal surgical resection followed by concomitant chemoradiotherapy and adjuvant chemotherapy (TMZ), patient outcomes remain nearly universally fatal [7]. The five-year relative survival rate following a GBM diagnosis is 6.8%, much lower than that for malignant CNS tumors (35.8%) and non-malignant CNS tumors (91.5%) [8]. Given the poor survival rates with currently approved GBM treatments, new therapeutic strategies are urgently needed.

GBM is characterized by high intratumoral heterogeneity, lack of immunogenicity, presence of GSCs, existence of the blood–brain barrier (BBB), and resistance to conventional therapeutics [9–13]. The treatment of the disease is mainly based on alleviation of symptoms and palliative care. Palliative care is generally used to improve the patient's quality of life and to give the patient the longest possible time to live. It includes surgery, radiation therapy and chemotherapy. Surgery is generally used to remove as much of the tumor as can be removed, along with focused radiation and chemotherapy. Temozolomide chemotherapy today belongs to the standard of care for glioblastoma, and there is evidence that temozolomide makes tumor cells more sensitive to radiation therapy [14, 15]. The therapeutic benefit of temozolomide depends on its ability to methylate DNA which damages the DNA and triggers the death of tumor cells. However, some tumor cells could decline the therapeutic efficacy of temozolomide by expressing a protein O6-alkylguanine DNA alkyltransferase (AGT) which could repair this type of DNA damage [16–18]. For temozolomide usage in GBM therapy, it is initiated first in combination with radiotherapy and subsequently as maintenance therapy. Several novel therapies are being developed to address the challenges in GBM treatment and minimize adverse effects [6]. For example, immune checkpoint inhibitors (ICIs), chimeric antigen receptor T-cell (CAR-T) therapy, targeting wild-type IDH enzymes, vaccine therapies, focused ultrasound therapy, oncolytic virotherapy, and novel bioengineered nanoparticles, including exosomes, are all in the pipeline for GBM treatment [2, 10, 19–25].

Eukaryotic aerobic respiration is the main energy supply pathway and is divided into three phases: anaerobic glycolysis, the tricarboxylic acid cycle (TCA cycle), and oxidative phosphorylation. Glycolysis, which does not require oxygen, is accomplished in the cytoplasmic matrix, while the latter two phases are carried out in the mitochondria. Pyruvate molecules produced by glycolysis are actively transported across the inner mitochondrial membrane and into the matrix where they are oxidized to form acetyl coenzyme A (Acetyl-CoA), the “fuel” of the TCA cycle, which ultimately produces ATP, GTP, NADH and FADH2 during the cycling of intermediates such as oxaloacetate (OAA). The production of ATP from glucose and oxygen during aerobic respiration is about 13 times higher than during anaerobic respiration [26]. Tumor cells require ample ATP to synthesize bioactive compounds such as lipids, proteins, and nucleotides for rapid proliferation [27]. The majority of ATP in tumor cells is generated via the oxidative phosphorylation (OXPHOS) pathway. Interference with OXPHOS cause cell cycle arrest suggesting that mitochondria play a role in cell proliferation [28].

In mitochondrial OXPHOS high-energy products are oxidized to release energy for the synthesis of ATP, and enzyme complexes on the inner mitochondrial membrane mediate this process. Under abnormal conditions, ROS are generated from oxygen that is instead prematurely and incompletely reduced [29]. Another source of ROS production in animal cells is the electron-transfer reaction catalyzed by the mitochondrial P450 system in steroidogenic tissues [30]. These P450 systems depend on the transfer of electrons from NADPH to P450. During this process, some electrons “leak” and react with O2 to produce superoxide. Steroidogenic tissues, ovaries, and testes contain large amounts of antioxidants, such as vitamin C (ascorbic acid) and beta-carotene, and antioxidant enzymes [31]. A variety of exogenous stimuli can also contribute to the formation of ROS, such as pollutants, heavy metals, tobacco, smoke, medications, exogenous substances, microplastics, or radiation.

Under normal physiological conditions, cells control ROS levels by balancing the production of ROS with the elimination of scavenging systems. However, excessive ROS can damage cellular proteins, lipids, and DNA, leading to lethal lesions in the cell, which in turn can lead to carcinogenesis. ROS are a double-edged sword. On the one hand, at low levels, ROS promote cancer cell survival because ROS are required to activate cell cycle processes driven by growth factors and receptor tyrosine kinases (RTKs) [32]. On the other hand, high levels of ROS can inhibit tumor growth by sustained activation of cell cycle inhibitors and by disrupting macromolecules to induce cell senescence and cell death [33, 34]. In normal cells, elevated levels of ROS are considered carcinogenic and lead to damage to biological structures, especially DNA [35]. On the contrary, increased ROS in tumor cells have tumorigenic effects, inducing various forms of cell death [36] Whether ROS signal survival or apoptosis in cancer cells is controlled by the dose, duration, type, and site of ROS production. In summary, cancer cells need moderate levels of ROS to survive, while too much ROS will kill them. ROS is thought to be a mediator of tumor cell eradication by radiotherapy [37, 38]. In fact, most chemotherapeutic and radiotherapeutic agents kill cancer cells by increasing ROS stress [39, 40]. Upregulation of ROS has been shown to enhance the efficacy of radiotherapy [41]. Enhancement of cellular oxidative stress with chloroquine in GBM therapy may increase radiotherapy-induced oxidative stress and thus improve therapeutic efficacy [40].

Mitochondria are key cellular organelles essential for various functions, including the regulation of cellular metabolism, redox signaling, calcium and ionic homeostasis, iron metabolism (biosynthesis of heme and iron-sulfur clusters), and cell death [42, 43]. Due to their fundamental roles in processes such as ATP production for cellular maintenance, ROS generation, and execution of cell death pathways, studying the role and regulation of mitochondria in GBM cells is of primary concern [44]. Mitochondria are widely associated with cancer, contributing to malignant transformation, tumor progression, and targeted therapy [45]. In cells, mitochondria are connected by a dynamic network that undergoes structural alterations through mitochondrial dynamics, which are balanced by the highly regulated processes of mitochondrial fission and fusion [46]. GBM cells often utilize alternative energy pathways in the absence of glucose, particularly deriving energy from fatty acids, leading to enhanced ROS output [42].

**Fig. 1:**
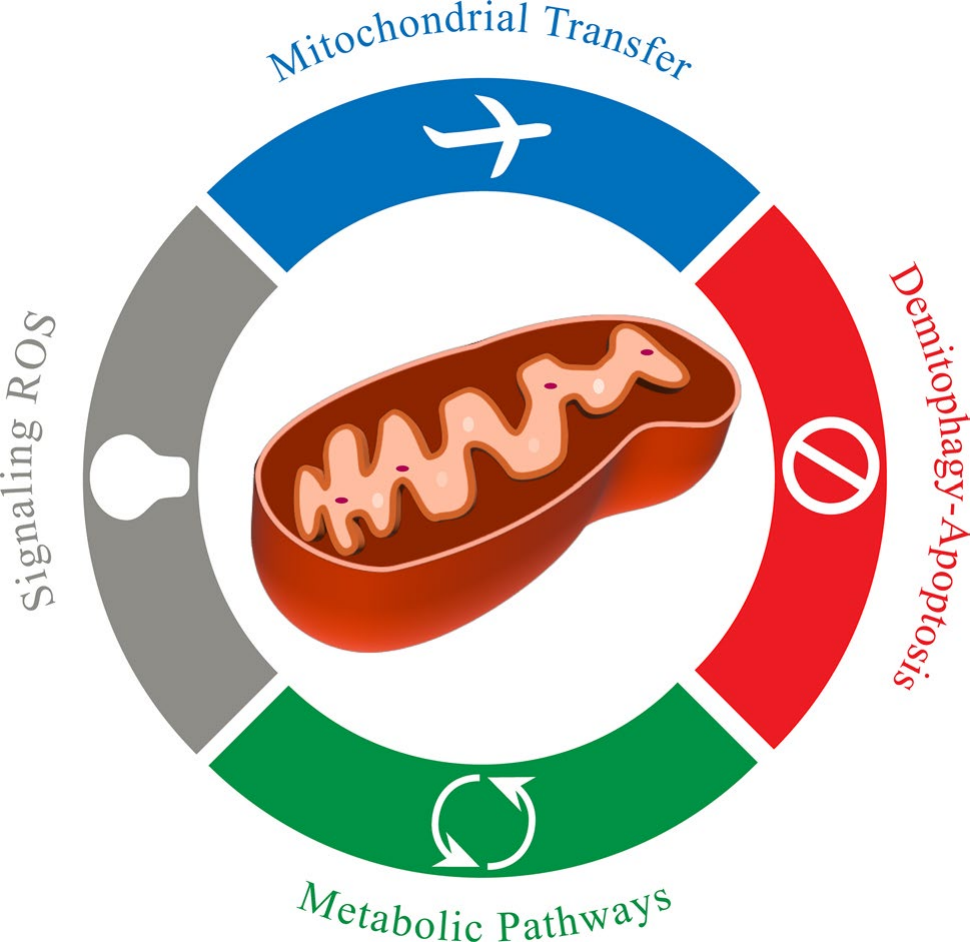
Potential mechanisms of targeting mitochondria: we suggest four principal topics in mitochondria that are related to targeted therapy for GBM therapy

With technological advancements and a deeper understanding of mitochondrial biological processes, new methods for treating glioblastoma, including mitochondrial-targeted therapies, are being explored. In this review, we discuss potential mitochondrial targets in GBM therapy and summarize the pathophysiological mechanisms of GBM mitochondria (Fig. 1).

## Metabolic pathways within mitochondria

**Fig. 2:**
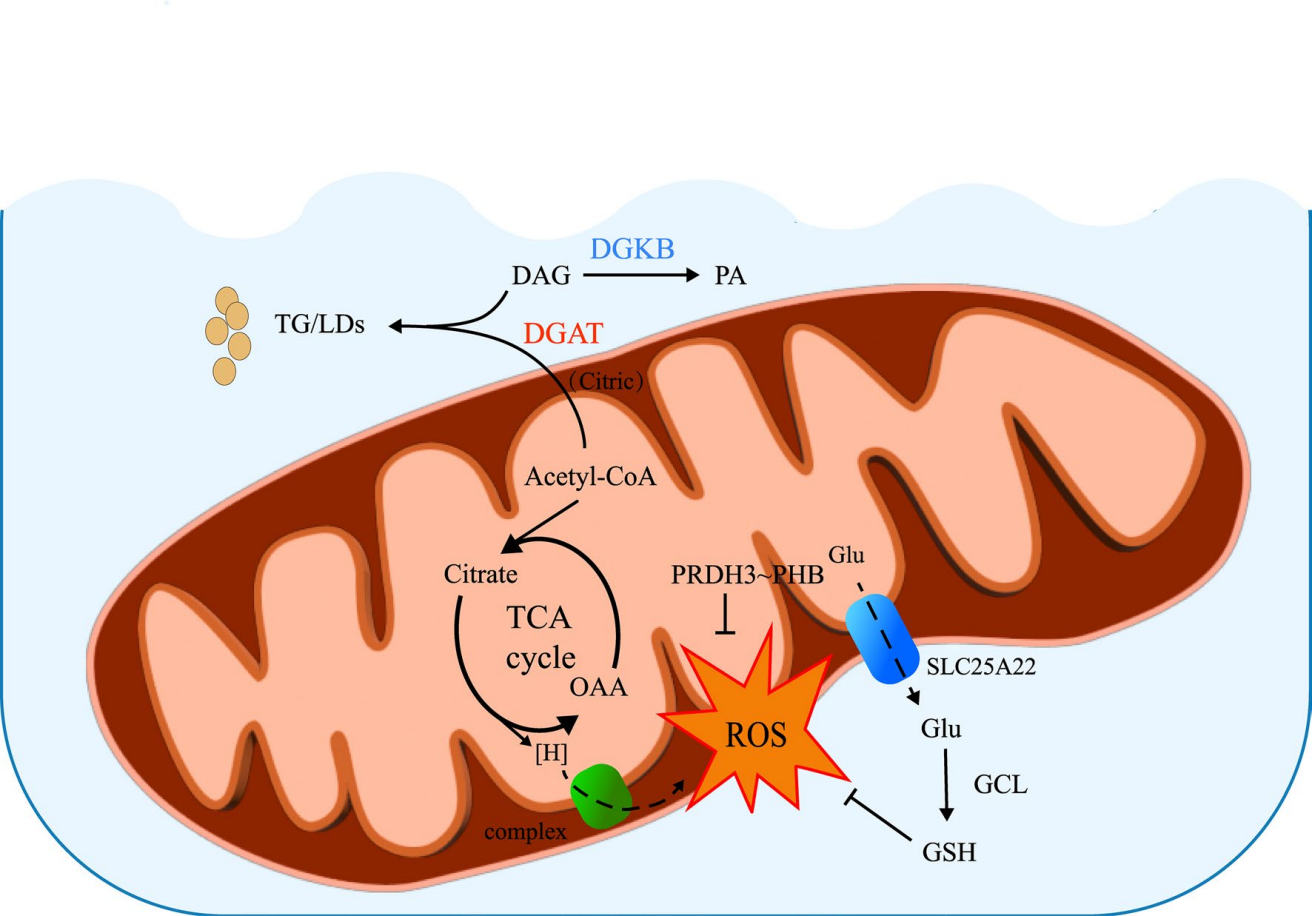
The molecular mechanism of mitochondrial metabolism depicted in the figure encompasses the pathways of lipid and glutamic acid metabolism, as well as ROS regulation in GBM. ROS, acting as a cytotoxic by-product of metabolism, significantly contributes to radiotherapy resistance in glioblastoma. The primary source of ROS is the electron transport chain (ETC) within mitochondrial oxidative phosphorylation (OXPHOS), where reduced hydrogen (primarily NADH, FADH2) is released across the ETC complex. This reduced hydrogen originates from the TCA cycle, with its substrate acetyl-CoA able to translocate into the cytoplasm as citric acid through the mitochondrial membrane and be stored in the form of triglycerides (TG) and LDs alongside DAG, a process regulated by diacylglycerol-acyltransferase (DGAT). Additionally, the content of DAG is modulated by diglyceride kinase β (DGKB). Glutathione, a critical cellular component regulating ROS levels, is primarily synthesized from Intracytoplasmic glutamate under the control of glutamate cysteine ligase (GCL). SLC25A22, a glutamate transporter spanning the mitochondrial membrane, facilitates the provision of raw material for glutathione (GSH) synthesis. Moreover, ROS regulation is contingent upon peroxiredoxins (PRDX), a ubiquitous family of antioxidant enzymes, with peroxiredoxin 3 (PRDX3) in GSCs being regulated by prohibitin (PHB)

Mitochondrial metabolism was once considered inconsequential in cancer therapy until the discovery of aerobic glycolysis, or the Warburg effect, which characterizes cancer cells' preference for glycolysis over mitochondrial oxidative phosphorylation [47]. Aerobic glycolysis is a hallmark of metabolic reprogramming in cancer cells, leading to the production of excess glycolytic intermediates and the accumulation of lactate. These metabolic changes are associated with tumor progression and resistance to antitumor therapies [48]. Consequently, targeting mitochondrial metabolism has emerged as a promising strategy to inhibit tumor progression [49]. The functional reinvention of mitochondria and the shift towards aerobic glycolysis are now recognized as hallmarks of cancer [50]. Mitochondria are the primary site of cellular function. Sugars, fatty acids, and amino acids enter the TCA cycle by conversion to acetyl coenzyme A as a substrate, which releases reduced hydrogen, which in turn enters the inner mitochondrial membrane for oxidative phosphorylation to generate ATP function. ROS, which may be generated in oxidative phosphorylation due to an inappropriate reaction with oxygen, can be regulated by glutathione (GSH) synthesized in the cytoplasm. Excess Acetyl-CoA can be transferred to the cytoplasm and stored with diacylglycerol (DAG) as lipid droplets (LDs). Diglyceride kinase B (DGKB), a regulator of intracellular concentrations of DAG, converts DAG to phosphatidic acid (PA) and regulates the corresponding levels of both lipids, playing a key role in cellular processes [51]. In tumor cells, high expression of the glutamine transporter, system ASC amino acid transporters 2 (ASCT2), allows for the cellular uptake of increased amounts of glutamine. The Glutamine is deaminated by glutaminase (GLS) in mitochondria to generate glutamate, eventually participating tricarboxylic acid cycle in the form of α-ketoglutarate [52, 53].Beyond glycolysis, which is the dominant energy source, several other metabolic pathways play crucial roles in glioblastoma metabolism. These include fatty acid oxidation, the glutamine metabolism, the metabolism of branched-chain amino acids, and mitochondrial oxidative phosphorylation. Each of these pathways may serve as potential therapeutic targets for future research and development (Fig. 2).

## Lipid metabolism

Compared to carbohydrate metabolism, fatty acids offer a higher energy density to cancer cells. Enzymes associated with fatty acid oxidation (FAO) are upregulated in GBM tissue [54]. Mitochondrial aerobic respiration in GBMs has been experimentally linked to FAO activity [54, 55]. Additionally, enzymes and functional proteins involved in fatty acid oxidation are elevated in GBMs [54, 56]. Moreover, inhibiting FAO results in reduced viability and increased apoptosis in GBM cells [56]. Acyl-CoA-binding protein (ACBP), also known as DBI, a neural stem cell pro-proliferative factor, has been identified as a regulator promoting FAO in GBM. Its therapeutic vulnerability has been uncovered in subsequent studies [57–59]. ACBP may additionally supply energy for tumor cell biosynthesis and metabolism by enhancing FAO metabolism in GBM cells. We have found the high expression of ACBP in GBM and that ACBP expression is important for GBM cell cycle regulation, and lack of ACBP hinders tumor proliferation and affects mouse survival [60]. Unfortunately, the tumorigenesis function of ACBP in GBM does not appear to be mediated through γ-aminobutyric acid (GABA) signaling, and evidence exists that this function is associated with fatty Acyl-CoA binding [57, 59]. Drugs that inhibit the FAO pathway such as etomoxir are not in clinical use for the time being due to hepatotoxicity [61]. The development of specific ACBP-targeted antitumor therapies holds great promise.

Diacylglycerol-acyltransferase 1 (DGAT1) is an enzyme responsible for storing acetyl-CoA in the form of triglycerides (TG) and lipid droplets (LDs) with the assistance of DAG. Upregulation of DGAT1 has been observed in GBM, enabling the storage of fatty acids in TG and LDs to prevent their entry into mitochondria and subsequent ROS generation thus giving GBM radioresistance [62]. The therapeutic significance of cladribine, a clinical drug that activates DGKB in vitro and in vivo, inhibits DGAT1, and sensitizes GBM cells to radiotherapy, has been demonstrated in animal studies [63]. Another study demonstrated that DGKB, a regulator of intracellular DAG concentration, catalyzes the conversion of DAG to PA to prevent Acetyl-CoA storage. DGKB was found to be downregulated in radioresistant GBM cells, while DGAT1 increased following ionizing radiation (IR) exposure, both mechanisms reducing acetyl-CoA entry into mitochondria and ROS production [62]. Thus, DGKB and DGAT1 present potential therapeutic targets to overcome GBM radioresistance, particularly considering the metabolic reprogramming observed in irradiated GBM tumors [64].

## Amino acid metabolism

Amino acids serve as primary substrates for biosynthesis in mammalian cells, with an even greater demand in tumor cells due to their heightened energy metabolism levels [65]. Glutamine is an important metabolic fuel that helps rapidly proliferating cells meet the increasing demand for ATP, biosynthetic precursors and reducing agents. Glutamine synthesis from glutamate and ammonia is catalyzed by glutamine synthetase. The majority of glutamine production occurs in muscle tissue, accounting for approximately 90% of all synthesized glutamine [66]. Despite being nonessential amino acids that can be synthesized from glucose in normal cells, many malignancies exhibit an increased requirement for glutamine [67]. In highly proliferating cells, glutamine consumption can exceed the amount needed for protein synthesis by as much as ten-fold, with cultured tumor cells necessitating at least ten times more glutamine than any other amino acid [68, 69]. In addition to nitrogen, glutamine provides mitochondria with precursors for maintaining mitochondrial membrane potential and synthesizing nucleotides, proteins, and lipids. In 1971, Kovacevic and colleagues observed that the carbon from glutamine ends up as carbon dioxide released by the cell, suggesting that glutamine may serve as a mitochondrial respiratory substrate [70]. This dependence of cancer cells on glutamine and its products has been termed “glutamine addiction”, which makes targeting glutamine metabolism an attractive therapeutic target for anticancer therapy [55, 67, 71].

Glutamine enters and exits cells via bidirectional transport mediated by the transporter proteins SLC1A5, orASCT2 and SLC7A5, which are involved in the activation of the mammalian target of rapamycin (mTOR) pathway to stimulate cell growth and also to inhibits autophagy [52, 72]. Glutamine is converted to glutamate in mitochondria in a deamination reaction catalyzed by GLS, and glutamate is converted by glutamate dehydrogenase (GDH) to α-ketoglutarate (α-KG), an intermediate of the TCA cycle. GLS 1 is highly expressed in many tumors and promotes tumor proliferation, and its high expression is thought to be regulated by transcriptional activation of the proto-oncogene MYC [73]. Mutational inhibitors of GLS 1 have shown promise in preclinical models of cancer, and a potent compound in this class, CB-839, has entered clinical trials [73, 74]. It has been shown that CB-839 inhibits cell proliferation in GBM, and modifies key metabolites [75]. It also reported that Tamoxifen and raloxifene suppress the proliferation of breast cancer cells by targeting SLC1A5 [76]. In the cytoplasm, glutamate is converted by glutamate cysteine ligase (GCL) to produce glutathione (GSH), which protects cells against IR-induced ROS. Mitochondrial glutamate transporter SLC25A22 has been linked to GBM radioresistance by transporting glutamate from mitochondria to the cytosol and promoting GBM invasive phenotypes through proline-induced extracellular matrix (ECM) remodeling [77].

Branched-chain amino acids (BCAAs) play a distinct role in tumor metabolism, contributing to tumor metabolism reprogramming and various tumor phenotypes [78, 79]. In the WHO's 2021 novel classification of central nervous system (CNS) tumors, IDH-wildtype diffuse gliomas are recognized as GBM [4]. IDHWT GBM exclusively expresses high levels of branched-chain amino acid transaminase 1 (BCAT1), which initiates the catabolism of BCAAs [80]. Consequently, BCAT1 and the associated metabolic pathway represent promising targets for IDHWT GBMs [81]. BCAT1 expression is contingent on the α-ketoglutarate substrate concentration in GBM cell lines [80]. However, co-treatment with the BCAT1 inhibitor gabapentin and α-ketoglutarate results in synthetic lethality, mechanistically disrupting mitochondrial homeostasis by increasing the NAD^+^/NADH ratio [82, 83].

## Oxidative phosphorylation

Metabolic reprogramming stands as a well-recognized hallmark of cancer, significantly enriching treatment strategies for GBMs [84, 85]. As multifunctional organelles crucial to cellular metabolism, mitochondria play pivotal roles, including OXPHOS. Despite cancer cells predominantly relying on glycolysis for lactate production even in the presence of oxygen, OXPHOS remains integral to tumor progression, impacting the TCA cycle, generation of ROS, maintenance of membrane potential, and regulation of mitochondrial morphology [86].

Pharmacological inhibition of the electron transport chain (ETC) (Complex I–IV) or F1F0 ATP synthase effectively suppresses OXPHOS, thus inhibiting GBM progression [87]. Gboxin, a recently identified small molecule, selectively hampers GBM growth by targeting the activity of F0F1 ATPase complex V, sparing embryonic fibroblasts or neonatal astrocytes [88]. To enhance the applicability of Gboxin, a biomimetic nanomedicine (HM-NPs@G) was engineered to overcome barriers such as poor blood circulation, the blood–brain barrier (BBB), and nonspecific uptake by GBM tissue/cells, rendering it a promising treatment modality for GBMs [89].

## ROS acts as a signaling molecule

ROS are primarily generated by the mitochondrial electron-transport chain during normal metabolism and serve as significant secondary messengers in mitochondrial signal transduction [90, 91]. Excessive ROS levels are commonly considered a risk factor for tumors due to their DNA, protein, and lipid toxicity, which can lead to genetic material alterations. Cells regulate ROS levels through enzymes such as superoxide dismutase (SOD), catalase, peroxiredoxins, and glutathione peroxidases [41]. ROS accumulation induces oxidative stress, resulting in pathological defects [92]. However, in tumor cells, high proliferation rates and active metabolism lead to an overproduction of ROS, which is associated with tumor formation and progression [93]. In fact, tumors require appropriate levels of ROS to promote proliferation. Previous experiments demonstrated that loss of PINK1 is a driver of GBM biology, which leads to increased proliferation, decreased oxygen consumption, and increased glycolysis. Reduction of PINK1 expression leads to HIF1A stabilization via elevation of ROS [94].

Mitochondrial ROS are crucial for the proliferation of cancerous cells driven by K-ras oncogenes [95, 96]. Furthermore, ROS targets tumor-associated signaling pathways such as NF-kB, PI3K, and MAP kinase [97–100]. Nonetheless, downregulation of ROS has been observed in radiotherapy-resistant GBM cells, particularly in glioma stem cells (GSCs), promoting GSC self-renewal and therapeutic resistance [101]. Mitochondrial ROS containment is regulated by prohibitin (PHB), which stabilizes PRDX3, a mitochondrion-specific peroxidase, in GSCs [41]. Additionally, a cooperative interaction between the mitochondrial chaperone TNF-receptor-associated protein-1 (TRAP1) and the major mitochondria deacetylase sirtuin-3 (SIRT3) in GSCs has been identified. TRAP1 is the mitochondrial-dedicated hsp90 family member, chaperoning ETC complex to mediate regulation of mitochondrial respiration [102]. Increased expression of TRAP1 in GSC increases aerobic respiration while decreasing respiration-induced ROS generation, which is conducive to the maintenance of cellular stemness in GSC [103]. We hypothesize that this may be related to the chaperoning of ETC by TRAP1, and the exact mechanism needs to be further investigated. SIRT3 is a NAD-dependent deacetylase localized to mitochondria [104]. Cells deficient in Sirt3 exhibit metabolic alterations, including a significant increase in mitochondrial superoxide levels upon exposure to IR, suggesting that SIRT3 may have a similar ROS-regulating effect as TRAP1 [105, 106]. TRAP1 acts as a chaperone to stabilize the SIRT3 protein, and SIRT3 reduces ROS production in GSC through deacetylation and SOD2 activation [103]. The ROS scavenging system is not only essential for GSC to remain stemness but also relevant for treatment resistance to GCS radiotherapy and chemotherapy [101, 107].This complex confers metabolic plasticity to GSCs and facilitates their adaptation to nutrient deficiencies [103]. Therefore, the study of targeting drugs for these proteins has a promising future for in the treatment of GBM.

## Inhibition of mitophagy induces apoptosis

Mitochondrial autophagy or mitophagy is the process of selectively degrading damaged mitochondria frequently in response to imposed stresses, such as hypoxia and nutrient deprivation. It plays a key role in mitochondrial quality control and maintenance of mitochondrial and cellular homeostasis [108]. Dysfunction in mitochondria is implicated in various diseases, including neurodegenerative diseases, cardiovascular diseases, and cancer [109]. To preserve intracellular environment homeostasis and maintain mitochondrial network stability, the autophagy mechanism eliminates dysfunctional mitochondria. There exists a delicate balance between mitophagy and apoptosis, two distinct self-destructive processes mutually regulated within the same cell [110, 111]. Mitochondrial autophagy also eliminates healthy mitochondria to reduce overall mitochondrial mass as an adaptive response to stresses such as hypoxia and nutrient deprivation to ensure efficient utilization of scarce metabolites and oxygen, and again, to limit excess ROS production [112]. Cancer cells, characterized by heightened ROS levels, adapt to oxidative stress through mechanisms including mitophagy regulation to evade apoptosis [113, 114]. Damaged mitochondria, inefficient in executing oxidative phosphorylation (OXPHOS), result in transmembrane potential dissipation [115]. Inhibition of mitophagy leads to pathological ROS accumulation, accelerating mitochondria-mediated apoptosis [43, 110, 116]. Mitochondrial autophagy has now been shown to contribute to carcinogenesis, cell migration, iron death inhibition, cancer stemness maintenance, tumor immune escape, and drug resistance during cancer progression. However, many studies have also shown that in tumor cells, mitochondrial autophagy induces normal cellular metabolism and prevents cellular stress response and genomic damage by reducing damaging ROS, thereby inhibiting tumor progression accompanied by these changes [117]. Thus, due to the dual roles of mitochondrial autophagy in tumors, the role of mitochondrial autophagy in tumors remains controversial.

Recently, characterized mitophagy pathways, some of which are relevant to GBM, hold promise for its treatment [118]. The interaction between pyruvate kinase M2 isoform (PKM2) and Bcl2, phosphorylated by PKM2, promotes apoptosis with chaperone protein HSP90α1. A phosphorylation-deficient Bcl2 T69A mutant sensitizes GBM cells to oxidative stress-induced apoptosis, suggesting the HSP90-PKM2-Bcl2 axis as a potential therapeutic target in GBM intervention [116]. Another study showed that Linc00942 promotes SOX9 expression by interacting with TPI1 and PKM2, which facilitates temozolomide resistance, which may be related to the previously reported protective effect of PKM2 against oxidative stress, and the exact mechanism remains to be further study [119]. Furthermore, mitophagy inhibition induced by TRPML, a lysosomal cationic channel, triggers apoptosis, significantly suppressing GBM growth both in vitro and in vivo [110]. Silencing NIX enhances GBM survival under hypoxia via NFE2L2/NRF2 transactivation-induced mitophagy [120]. SFN-Cys decreased mitophagy protein Bnip3 and Nix, and disrupted mitochondria morphology, indicating SFN-Cys might also inhibit mitophagy. This may offer a novel therapeutic strategy against migration and invasion in GBM [121].

Understanding the molecular regulation mechanism of mitophagy not only deepens our understanding of various diseases but also identifies new drug targets and advances clinical treatments. Targeting obstacles in mitophagy pathways holds therapeutic potential in cancer treatment, leveraging the cytotoxic effects of damaged mitochondria for tumor clearance. Hence, studying mitophagy is pivotal in broadening our understanding of mitophagy-related disease pathogenesis and offers a promising avenue for drug development, potentially offering solutions for GBM treatment.

## Diverse modulatory effects of mitochondrial transfer

As the primary powerhouse for energy metabolism and other cellular processes, mitochondria are susceptible to damage and morphological changes, undergoing alterations in location. Physiologically, mitochondrial reprogramming and axonal transport play significant roles in maintaining mitochondrial homeostasis [122, 123]. Dysfunctional mitochondria in normal cells produce excessive cytotoxic ROS due to a lack of ROS scavenging mechanisms. However, the association of mitochondrial transfer with this phenomenon remains inconclusive [124].

Research on mitochondrial movement within and between cells has garnered considerable interest. The mitochondria transfer mechanisms was divided into three categories: transient cellular connections for mitochondria, ejection of mitochondria in extracellular vesicles (EVs) and release of free mitochondria for capture. Besides supporting metabolism of recipient cell and donor cell mitochondria quality control, mitochondria transfer also acts as a regulator in metabolic homeostasis an immune system. Mitochondrial transfer impacts various aspects of cancer, including basic mitochondrial functions, cancer cell survival and proliferation, tumorigenesis, tumor progression, and chemoresistance [125–130]. Importantly, mitochondrial transfer has been demonstrated between different cells in the central nervous system, suggesting a role in cell–cell signaling within the tumor microenvironment (TME) [131, 132]. This process may contribute to tumorigenicity and therapy resistance through two major connections in GBM: (1) Tumor microtubes, ultra-long membrane protrusions extended from tumor cells in vivo, (2) Tunneling nanotubes (TNTs), thin membrane tubes connecting distant cells and facilitating the transfer of cellular content [133–138].

Growth-associated protein 43 (GAP43) is a significant structural protein of tumor microtubes and is important for the formation of TNTs, particularly in GBM. Mitochondrial transfer from astrocytes to GBM, facilitated by GAP43, promotes proliferation and tumor growth [129]. MiR-185-5p and lncRNA vof16 were effective in targeting GAP43 in spinal cord transection (SCT) rats, which may inhibit neurite growth and axonal growth [139]. The effectiveness of RNA-based targeted drugs in glioma treatment needs to be further validated. However, co-incubation of mitochondria from normal human astrocytes with GBM cells inhibits malignant proliferation and enhances radiosensitivity in GBM cells [140]. Presumably, mitochondria transferred from astrocytes in GBM patients may exacerbate the condition due to their source. Thus, targeting mitochondrial transfer pathways holds promise as a treatment strategy, although the mechanisms underlying these disparate results require further exploration.

## Conclusion

This review delved into recent advancements in mitochondrial pathways and their physiological processes concerning glioblastoma. By citing examples from existing experiments, potential therapeutic targets associated with mitochondria were introduced. Thus, mitochondrial metabolism, encompassing branched-chain amino acids (BCAAs) and oxidative phosphorylation (OXPHOS), regulation of ROS, mitochondrial transfer, and mitophagy-induced apoptosis, have shown promising outcomes in basic research. While most mitochondrial therapies have yet to transition into clinical practice for cancer treatment, significant strides have been made in their development. Other emerging therapeutic modalities, such as oncolytic viruses and exosomes, are also under investigation, although further validation is required before their clinical application.

Considering the heterogeneous nature of glioblastoma and the challenges posed by current therapies, the advancement of mitochondria-targeted therapy methods holds potential for significant progress in the treatment of this disease.

## Data Availability

No datasets were generated or analysed during the current study.
